# Interfacial Speciation Determines Interfacial Chemistry: X‐ray‐Induced Lithium Fluoride Formation from Water‐in‐salt Electrolytes on Solid Surfaces

**DOI:** 10.1002/anie.202007745

**Published:** 2020-10-09

**Authors:** Hans‐Georg Steinrück, Chuntian Cao, Maria R. Lukatskaya, Christopher J. Takacs, Gang Wan, David G. Mackanic, Yuchi Tsao, Jingbo Zhao, Brett A. Helms, Kang Xu, Oleg Borodin, James F. Wishart, Michael F. Toney

**Affiliations:** ^1^ SSRL Materials Science Division SLAC National Accelerator Laboratory Menlo Park CA 94025 USA; ^2^ SLAC National Accelerator Laboratory Joint Center for Energy Storage Research (JCESR) Lemont IL 60439 USA; ^3^ Department Chemie Universität Paderborn 33098 Paderborn Germany; ^4^ Laboratory for Electrochemical Energy Systems Department of Mechanical and Process Engineering ETH Zürich 8092 Zürich Switzerland; ^5^ Department of Chemical Engineering Stanford University Stanford USA; ^6^ Department of Chemistry Stanford University Stanford USA; ^7^ Joint Center for Energy Storage Research Lawrence Berkeley National Laboratory Berkeley CA 94720 USA; ^8^ The Molecular Foundry Lawrence Berkeley National Laboratory Berkeley CA 94720 USA; ^9^ Energy Storage Branch Sensor and Electron Devices Directorate U.S. Army Research Laboratory Adelphi 20783 USA; ^10^ Chemistry Division Brookhaven National Laboratory Upton NY 11973 USA; ^11^ Department of Chemical and Biological Engineering University of Colorado Boulder CO 80309 USA

**Keywords:** aqueous lithium-ion batteries, interfaces, interphases, water-in-salt electrolyte, X-ray chemistry

## Abstract

Super‐concentrated “water‐in‐salt” electrolytes recently spurred resurgent interest for high energy density aqueous lithium‐ion batteries. Thermodynamic stabilization at high concentrations and kinetic barriers towards interfacial water electrolysis significantly expand the electrochemical stability window, facilitating high voltage aqueous cells. Herein we investigated LiTFSI/H_2_O electrolyte interfacial decomposition pathways in the “water‐in‐salt” and “salt‐in‐water” regimes using synchrotron X‐rays, which produce electrons at the solid/electrolyte interface to mimic reductive environments, and simultaneously probe the structure of surface films using X‐ray diffraction. We observed the surface‐reduction of TFSI^−^ at super‐concentration, leading to lithium fluoride interphase formation, while precipitation of the lithium hydroxide was not observed. The mechanism behind this photoelectron‐induced reduction was revealed to be concentration‐dependent interfacial chemistry that only occurs among closely contact ion‐pairs, which constitutes the rationale behind the “water‐in‐salt” concept.

## Graphical abstract

Using synchrotron X‐rays the LiTFSI/H_2_O electrolyte interfacial decomposition pathways in the “water‐in‐salt” and “salt‐in‐water” regimes are investigated. The resultant photoelectron‐induced reduction was revealed to be concentration‐dependent interfacial chemistry that only occurs among closely contact ion‐pairs, which constitutes the rationale behind the “water‐in‐salt” concept.

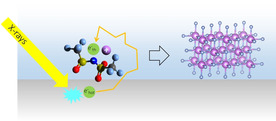

## Introduction

Despite the commercial success of lithium‐ion batteries (LIBs) in our life ranging from personal electronic devices, to electric vehicles and grid storage, they still raise significant safety concerns, owing primarily to the combination of their high energy density and the utilization of flammable non‐aqueous electrolytes. Although catastrophic failure events are rare, they carry significant human and economic impact, as evident by recent calamities involving both electronic devices and electric vehicles. Water‐based electrolytes offer an intrinsically safe (and potentially cheap) alternative, but typically they suffer from a narrow electrochemical stability window of only 1.23 V, which prevents aqueous high energy density LIBs to compete with their non‐aqueous counterparts operating at >4 V. Over the last few years, this issue of narrow voltage window was overcome via the ground‐breaking utilization of highly concentrated electrolytes, that is, 21 m lithium bis(trifluoromethanesulfonyl)imide (LiTFSI) in water and its later progenies.^[1]^ These fascinating systems are denoted water‐in‐salt electrolytes (WiSE), as salt outnumbers solvent (water) by weight or volume.^[2]^ Advantageous interphases and interfacial electrolyte properties are at the heart of their performance. Specifically, the chemistries appear to benefit from the formation of a passivating solid‐electrolyte‐interphase (SEI)^[3]^ on the anode and favorable inner‐Helmholtz layer structure on the cathode,^[4]^ which kinetically hinder hydrogen and oxygen evolution, that is, pushing their onset voltages to lower and higher potentials, respectively, thus widening the stability window to 3 V. Even further progress has been made by using a bi‐salt approach,^[5]^ hybrid aqueous/non‐aqueous,^[6]^ by designing active protection layers,^[7]^ reaching 4 V aqueous LIBs, as well as by extending the WiSE approach to acetate chemistries^[8]^ and zinc metal ion‐batteries.^[9]^

The fundamental understanding of WiSE and extension of electrolyte electrochemical stability is still limited and controversial, in particular regarding interfacial processes such as passivation layer formation and the role of anions in these phenomena.[^1a^, ^3^, ^10^] For instance, as the negative electrode is polarized beyond −1.0 V vs. SHE, H_2_ evolution contributed the majority of reduction current, leading to LiOH formation, LiTFSI precipitation and decomposition,[^10a^, ^11^] in accord with molecular dynamics (MD) simulation predictions of water enrichment at the negative electrode surface below −1.0 V compared to the bulk.[^4b^, ^12^] However, at low negative polarization of Pt electrode to −0.55 V vs. SHE (2.5 vs. Li/Li^+^), LiF was the major SEI component,^[5c]^ in accord with passivation of Mo_6_S_8_ anode.^[1a]^

In order to obtain additional insights into SEI composition, structure, and formation mechanism in WiSE, we performed in situ experiments using synchrotron X‐rays on the SEI in LiTFSI/H_2_O solutions, where ionizing X‐rays produce a reductive environment at the solid's surface via the generation of photoelectrons and secondary electrons, while simultaneously probing the structure of surface films formed via X‐ray diffraction. We denote these measurements as X‐ray chemistry‐X‐ray probe experiments (XCXP).^[13]^ This approach allows us to investigate in situ the structural properties of surface films without the necessity to remove the surface from its natural environment. Conceptually, these experiments are similar to experiments performed in the field of radiation chemistry, where ionizing radiation is utilized to produce transient species via excitation and ionization. The nature of these species and their chemical reactivity, as well as reaction products, are probed via a variety of methods.

Our results show that the photoelectron‐induced reduction of LiTFSI contact ion pairs leads to LiF formation, for which we propose a reaction mechanism. We hypothesize that SEI films could be improved if the observed direct reduction of TFSI^−^ towards LiF could be achieved electrochemically. Our experiments not only provide insight into the rational design of better aqueous interphases, but also expose the importance of circumventing artifacts from photoelectron‐induced reductive reactions when analyzing surface structure and composition using high‐intensity ionizing synchrotron radiation.

## Results and Discussion

**Figure 1:**
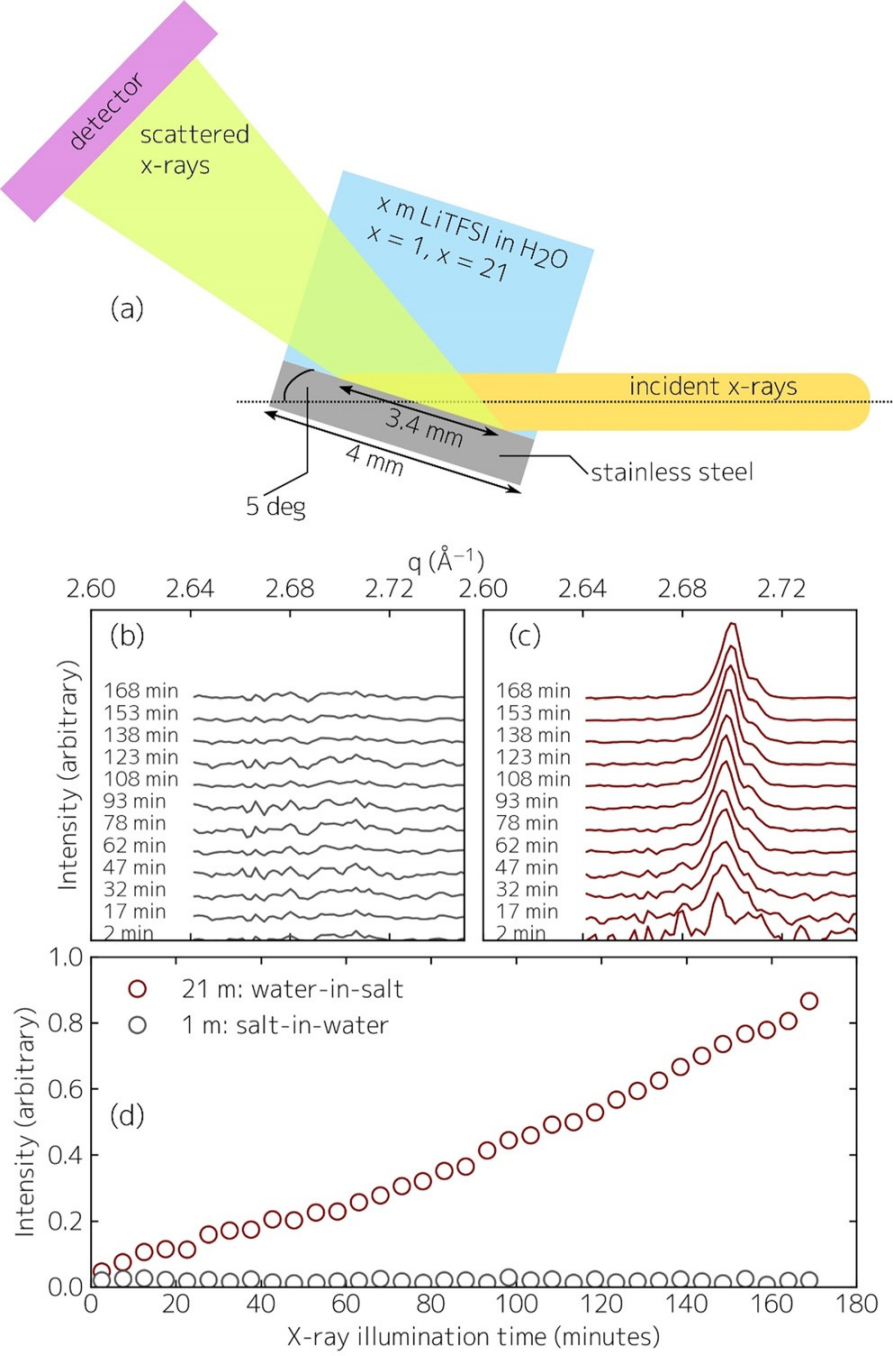
a) Experimental X‐ray‐chemistry X‐ray‐probe (XCXP) setup. b) Background‐subtracted LiF (111) X‐ray diffraction intensity as a function of X‐ray exposure time of the electrolyte/stainless‐steel interface for the 1 m salt‐in‐water solution. c) Same as (b) for the 21 m water‐in‐salt solution; the shoulder at the high‐q side of the LiF (111) peak corresponds to signal originating in the electrolyte/Kapton window interface. The curves are vertically shifted for clarity. d) Integrated LiF (111) intensity as a function of X‐ray exposure time.

Figure 1 a shows the experimental setup used for our XCXP experiments to unravel the interfacial chemistry of different concentration LiTFSI solutions in water (1 m *salt‐in‐water* solution and 21 m *water‐in‐salt* solution; the solutions were made from “Battery Grade” LiTFSI salt (Gotion) and >18 MOhm Millipore water). X‐rays impinge the solid–liquid interface at an incident angle of five degrees. At the vertical and horizontal beamsize used in our experiments, this corresponds to an exposed surface area of 3.4×1.0 mm=3.4 mm^2^. After sample alignment with heavily attenuated beam, the X‐ray shutter was opened for ≈180 minutes, and the surface was continuously irradiated while X‐ray diffraction patterns were collected for 30 seconds each (later averaged over five minutes) using a stationary area detector position that allowed for simultaneous collection of the LiF (111) and (200) peak, as well as several stainless steel peaks and the LiOH (110) peak.

Upon illumination of the surface with X‐rays, photoelectrons are generated from the absorbing material (details regarding the absorbed dose in Supporting Information). The majority of these interact with the substrate and/or solvent quickly, and produce secondary electrons upon thermalization (see below for a more in‐depth discussion). These processes result in substantial production of excess electrons at the surface, creating a reductive environment.^[14]^ Conceptually, this is similar to applying cathodic potentials to the stainless steel “electrode” in an electrochemical cell.

The scattering patterns as a function of time upon X‐ray illumination in the scattering vector range for the LiF (111) peak for selected times (averaged over 5 minutes) are shown in Figure 1 b for the aqueous 1 m and Figure 1 c for the WiSE 21 m solutions, respectively. The emergence of the LiF (111) peak at *q*=2.702 Å^−1^ and (200) peak (Figure S1) for the 21 m solution is clearly observed (Figure 1 c), while the scattering pattern for the 1 m solution remained unchanged and no diffraction peak appeared (Figure 1 b). This behavior is quantified in Figure 1 d, which illustrates the integrated LiF (111) intensity as a function of time, showing the growth of crystalline LiF for the WiSE 21 m solution, whereas no LiF growth was observed for the aqueous 1 m solution (this was also verified via XPS after the completed XCXP experiment, Figure S2). Interestingly, a faster than linear growth rate is observed. To exclude the possibility that absence of the LiF diffraction signal in the 1 m solution is a result of LiF formation and subsequent dissolution, the solubility of LiF in 1 m LiTFSI in H_2_O was measured. Since measured LiF solubility in 1 m LiTFSI is low (≈0.004 wt % as measured with an Apera Instruments, LLC WS100 Fluoride/pH Portable Meter Kit), we do not attribute the absence of LiF to dissolution. After disassembly of the cell, a white powdery film was visibly observed for the WiSE 21 m solution (more below). We note that we can rule out significant spontaneous LiF formation upon hourlong immersion of stainless steel in WISE, because XRD investigation of an unirradiated spot on the sample showed no LiF signal. Further, we point out that in XCXP, the X‐ray probe is only sensitive to crystalline electrolyte decomposition products; however, it has been shown via cryo‐TEM, albeit in other systems, that the SEI can also constitute amorphous phases.^[15]^ Given the relatively large thickness of the X‐ray‐induced interfacial LiF film in our cases and our compositional analysis showing mostly LiF (below), amorphous phases likely are of negligible presence under XCXP conditions.

The simplest explanation for the difference in LiF formation between the aqueous and WiSE systems is that the radiation chemistry of water dominates the interfacial radiolysis of the aqueous systems, while the water remaining in the WiSE electrolyte is fully complexed by the salt and is essentially a different chemical species. The primary radical products of water radiolysis (e^−^_aq_, H^.^ and ^.^OH) are relatively unreactive towards the uncomplexed TFSI^−^_aq_ anion. As discussed below, interfacial radiolysis in the WiSE system follows a different path.

The observed X‐ray‐induced interfacial LiF formation was also observed (using XCXP, XPS, and visual inspection) for WiSE solutions on sapphire (exemplary XCXP in Figure S3), silicon, platinum, silicon carbide, Kapton, PEEK, and PP, that is, metal, semi‐conductor, and insulator. This is consistent with the apparent non‐passivating growth, that is, LiF, initially nucleated on the stainless‐steel electrode, continues to grow on a different “substrate”, namely itself LiF. Accordingly, we conclude that X‐ray‐induced interfacial LiF formation occurs at any solid/WiSE interface. We note that we did not observe X‐ray‐induced LiF formation in bulk WiSE (Supporting Information).

**Figure 2:**
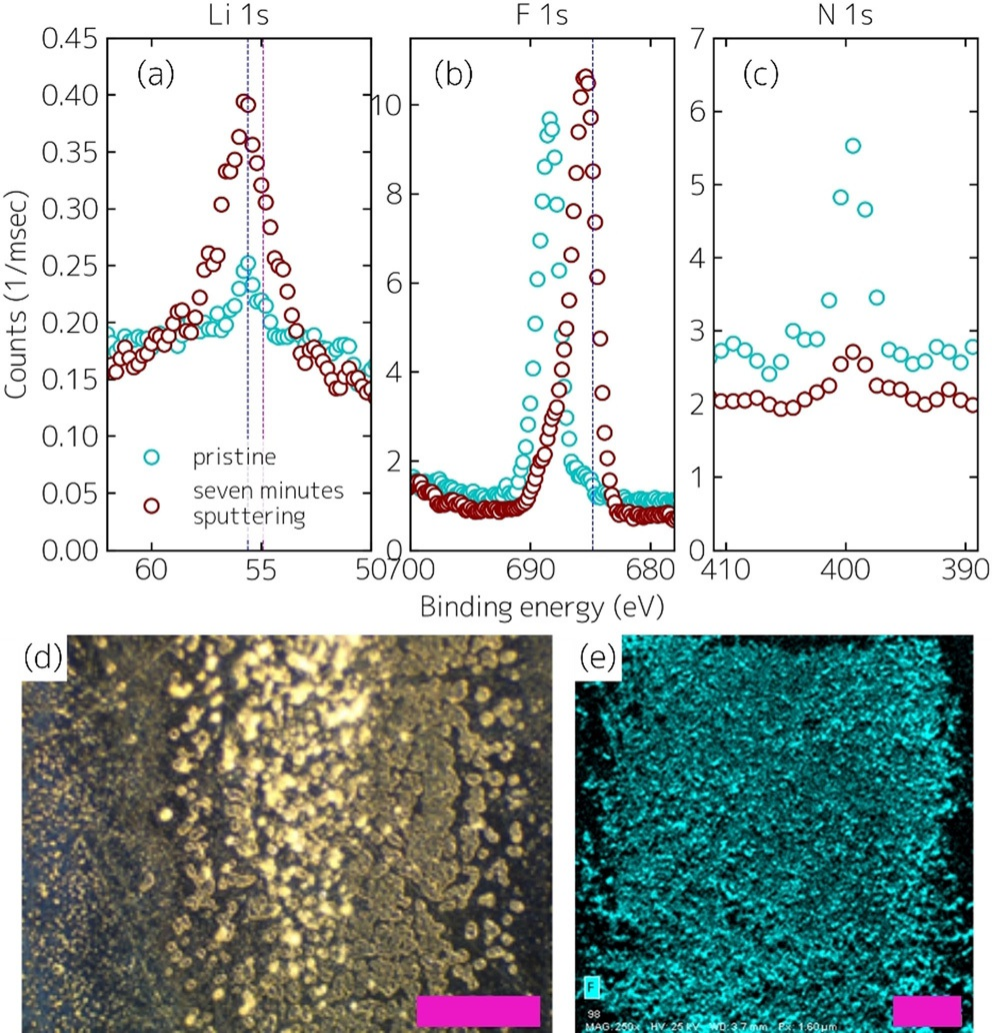
a) X‐ray photoelectron spectroscopy of stainless‐steel electrode after illumination with X‐rays in the solid–liquid interfacial scattering cell before (cyan) and after seven minutes of sputtering (red) in the Li 1s spectral range. b) Same as (a) in the F 1s spectral range. c) Same as (a) in the N 1s spectral range. The dashed blue lines correspond to the expected peak positions of LiF in the Li 1s spectral range (55.6 eV^[35]^) and F 1s spectral range (684.8^[36]^). The dashed magenta line corresponds to the expected peak position of LiOH (54.9 eV^[37]^); it is noted that at the resolution of our instrument LiF and LiOH are hard to distinguish. d) Optical micrograph of a sample prepared under the same conditions. Scale‐bar corresponds to 120 μm. e) SEM/EDX micrograph of a sample prepared under the same conditions. Scale‐bar corresponds to 100 μm.

In order to further investigate the surface film's morphology and chemistry formed upon X‐ray illumination, the WiSE 21 m samples were analyzed using ex‐situ X‐ray photoelectron spectroscopy (XPS), scanning electron microscopy energy‐dispersive X‐ray spectroscopy (SEM‐EDX), and optical microscopy. Prior to these experiments, the samples were rinsed with isopropanol. The XPS results before and after sputtering (2 kV, 1 μA for 2 min and 5 kV, 1 μA for 5 min) in the Li 1S, F 1s, and N 1s spectral ranges are shown in Figure 2 a–c. Before sputtering, remaining LiTFSI salt on the surface is observed (strong N signal), whereas after sputtering the main detected specie is LiF. XPS analysis shows a Li:F ratio of 50:50, with Li and F making up 82 % of the total signal, further confirming the presence of LiF as a dominant phase and that LiF constitutes the main decomposition product of TFSI^−^ decomposition under XCXP conditions. It must be noted that LiF can also be formed from LiTFSI as a result of Ar^+^ sputtering;^[16]^ however, in light of the XCXP evidence for LiF presented above, that is expected to be a minor effect here. We note that XPS on sapphire after XCXP yielded similar results, while XPS on immersed but unirradiated locations showed no LiF signal, further suggesting no spontaneous LiF formation upon hourlong immersion of solids in WiSE. An optical micrograph is shown in Figure 2 d demonstrating the existence of ≈10 μm large LiF agglomerates (in contrast to a smooth film). This is consistent with the non‐linear growth mode observed in XCXP, indicating that increased surface area upon growth leads to increased growth rate because the reaction occurs at any solid/liquid interface (the corresponding reaction mechanism is presented below). The SEM‐EDX image (Figure 2 e) provides a complementary picture regarding the morphology and shows significant presence of F in the film.

Combining all surface characterization results from XCXP, XPS, optical microscopy, and SEM‐EDX leads us to conclude that the X‐ray‐induced reaction layer formed at the 21 m WiSE/solid interface is mostly composed of LiF. In other words, we observed the X‐ray‐induced formation of LiF at the solid/WiSE interface. Specifically, X‐ray illumination creates a reductive environment by generation of electrons at the surface of the solid, and hence at high concentrations LiF formation is observed in 21 m LiTFSI/H_2_O solution. As fluorine is only present in the system as TFSI^−^, and Li^+^ is only present in its ionic form, the corresponding reaction must involve these two species, that is, a defluorination of TFSI^−^ or defluorination of its decomposition fragments if the S−N bond breaking occurs first. Furthermore, the reaction can only occur when TFSI^−^ is near the surface, and close to Li^+^, that is, under interfacial contact ion pair conditions. Formation of such positively charged (Li_2_TFSI)^+^ aggregates is essential for stabilization of an excess electron on (Li_2_TFSI)^+^ as a first step of reductive decomposition of the TFSI and by further bringing the positively charged cluster to the negative electrode. The latter is in agreement with previous reports.^[17]^ For 1 m LiTFSI, salt is completely dissociated and TFSI^−^ anions are repelled from the negative electrode. The full dissociation at low concentrations was shown via Fourier‐transformed infrared (FTIR) measurements,^[3]^ and MD simulations.[^4b^, ^17a^, ^18^]

Our observation that the photoelectron‐induced reduction reaction leading to the formation of LiF from LiTFSI/H_2_O solutions only occurs at high concentrations (i.e. *water‐in‐salt*) is also in accord with quantum chemistry (QC) calculations. Specifically, the calculated energetics are such that a Li_2_TFSI cluster reductively decomposes to a LiFLi cluster in the presence of an excess amount of electrons, that is, at reductive potentials.[^1a^, ^17b^] At high potentials (2–3 V vs. Li/Li^+^), this LiF formation reaction was suggested to be a slow but thermodynamically favorable one.^[1a]^ Additional DFT calculations using an accurate but computationally expensive G4MP2 composite methodology were performed on the larger more representative (Li_2_TFSI(H_2_O)_6_)^+^ clusters surrounded by implicit water. They showed that the TFSI^−^ anion defluorination occurs at 2.14 V vs. Li/Li^+^, while the S−N bond breaking and CF_3_‐detachment occur at lower reduction potentials <2 V vs. Li/Li^+^ as shown in Figure S10. Less reliable DFT calculations predicted that S−N bond breaking is slightly more favorable than Li‐F‐Li formation, however. Both of these reactions were also observed at the LiF passivated lithium in the highly concentrated LiTFSI in ethers on the LiF‐covered lithium metal.^[17b]^ Importantly, at lower electrode potential (<2 V vs. Li/Li^+^) water adsorption at the interface becomes more pronounced,[^4b^, ^12^] resulting in the enhanced water reduction. Our current observations suggest that the first scenario of the LiTFSI reduction at higher potentials (>2 V vs. Li/Li^+^) is realized during XCXP where LiF formation dominates without observable LiOH (Figures S8).

Together, this leads us to hypothesize the following two‐step reaction mechanism towards LiF formation in reductive environments:

(1a) $$\mathrm{Li}{}^{+}\;+\left(R\text{-}\mathrm{CF}{}_{3}\right){}^{-}\;+e{}^{-}\;\left(\sec.\;\mathrm{electrons}\right)\to \mathrm{LiF}+\left(R\text{-}\mathrm{CF}{}_{2}\right){}^{-}\mathrm{with}\;R=\mathrm{CF}{}_{3}\text{-}\mathrm{SO}{}_{2}\text{-}N\text{-}\mathrm{SO}{}_{2}$$

(1b) $$\left(R\text{-}\mathrm{CF}{}_{2}\right){}^{-}\;\to \;T\;+\;e{}^{-}\;\left(\mathrm{to}\;\mathrm{substrate}\right)$$

where T corresponds to further reaction products of TFSI^−^,

with the following sub‐reactions:

(2a) $$\left(R\text{-}\mathrm{CF}{}_{3}\right){}^{-}+\;e{}^{-}\;\to \;\left(R\text{-}\mathrm{CF}{}_{3}\right){}^{2-}$$

(2b) $$\left(R\text{-}\mathrm{CF}{}_{3}\right){}^{2-}\;\to \;\left(R\text{-}\mathrm{CF}{}_{2}\right){}^{-}\;+\;F{}^{-}$$

(2c) $$F{}^{-}\;+\;\mathrm{Li}{}^{+}\;\to \;\mathrm{LiF}$$

**Figure 3:**
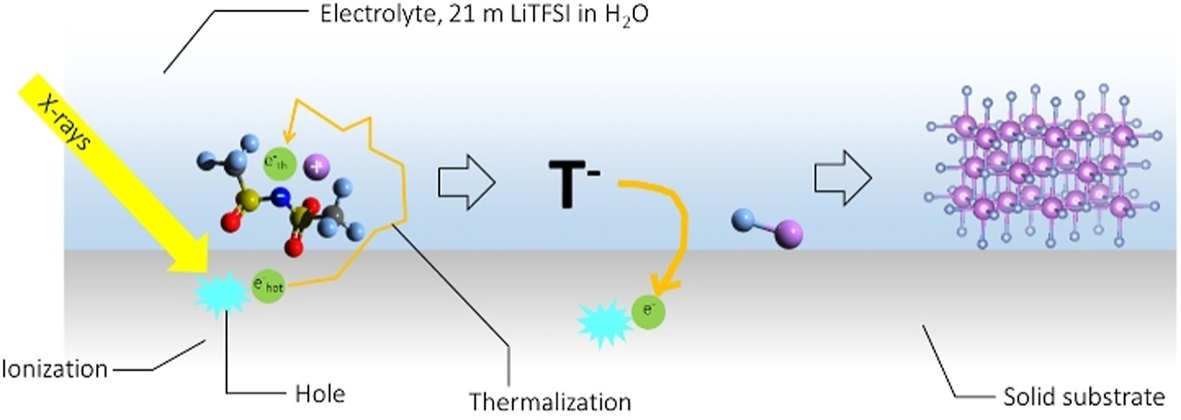
Proposed reaction mechanism for the reaction of Li^+^ and TFSI^−^ in WiSE system with an X‐ray‐induced photoelectron towards LiF formation. It is noted that this reaction occurs at any solid/WiSE interface under XCXP conditions. Molecules are drawn using Avogadro software.^[38]^

Here, an electron (primary photoelectron or secondary electron) attacks TFSI^−^ ((R‐CF_3_)^−^) and forms LiF and negatively charged defluorinated TFSI^−^ ((R‐CF_2_)^−^). In the sub‐reactions, the second negative charge on (R‐CF_3_)^2−^ represents the initial step of the reaction of the thermalized (pre‐solvated) or solvated electron near (R‐CF_3_)^−^ (discussed in detail below). Subsequently, the defluorinated TFSI^−^ is likely to react and give an electron back to the substrate, leading to charge neutrality after recombination with the initially formed hole. This also explains why the proposed reaction occurs universally on metals and insulators, as a reaction mechanism which does not involve electron transfer back to the substrate as it would lead to a significant amount of charging, unphysical for insulating substrates. We speculate that the defluorinated TFSI^−^ radical can form dimers (Figure S11), and possibly oligomers or polymers upon further defluorination. NMR spectroscopy (Figure S7) did not show any observable signal of a CF_2_ (or CHF_2_) group, which may be because the concentration is below the detection limit. Another possible reaction mechanism could involve reduction of TFSI^−^ towards SO_2_CF_3_ and LiNSO_2_CF_3_,^[17b]^ as found in soft X‐ray irradiation of TFSI^−^,^[19]^ and subsequent decomposition and defluorination of these species as found in simulations in highly reductive environments.^[20]^ The proposed mechanism is schematically illustrated in Figure 3; while it was inferred from predictions,^[1a]^ it in turn provides validation of these predictions.

Even though XCXP conditions mimic reductive environments, it is instructive to consider likely differences. While reductive radiation‐induced LiF formation and reductive electrochemically‐induced LiF formation (as in SEI growth during battery operation) proceed via the reaction mechanism of reductive TFSI^−^ decomposition, a crucial difference is the passivating behavior. In radiation‐induced LiF formation, the electron participating in the reduction reaction (reaction (1a)) is produced directly at the junction between solid and electrolyte via the photoelectric effect. In the pristine state, the solid is stainless steel, however, once LiF is formed, LiF represents the solid. Hence the film formation is not passivating, and LiF keeps growing even after 3 hours (Figure 2). On the contrary, in electrochemical LiF formation, the electron participating in the reduction reaction is produced in the working electrode and has to travel through the surface film to reach the solid/liquid interface. Accordingly, once the surface film is thick enough to block electrons, the surface is passivated. Since the only difference between radiation‐induced and electrochemical LiF formation via reductive TFSI^−^ decomposition is the origin of the electron, which does not affect the reaction mechanism, our results are similar to those occurring in realistic battery environments.

It must be noted in passing that while we did not observe any visible bubble formation during XCXP, concomitant electrolysis cannot be excluded. This could play a role as hypothesized by Dubouis et al.,^[10a]^ who proposed the formation of an electrochemically‐formed passivation layer from 21 m LiTFSI in H_2_O via the formation of OH^−^ during hydrogen evolution reaction. OH^−^ would subsequently react with Li^+^ to form precipitated LiOH and would participate in nucleophilic attack of TFSI^−^, in which the CF_3_ group is degraded and LiF is formed. However, in contrast to the electrochemical study by Dubouis et al.^[10a]^ we observe no LiOH during XCXP (Figures S8) or conclusive LiOH signal in ex‐situ XPS (Figure 2). This indicates the direct reduction of TFSI^−^ to form LiF during XCXP, in contrast to a water or OH^−^‐mediated decomposition mechanism, albeit at different experimental conditions. We speculate that unlike the water‐mediated mechanism, the direct TFSI^−^ reduction could lead to a different and potentially improved SEI formation and higher initial Coulombic efficiency. To conclude the discussion of the potential reaction mechanism occurring under reductive environments and their relation to our findings, we postulate that the direct reduction of LiTFSI towards LiF (reaction (1a)) is the main mechanism in XCXP environment, which consists of excess electrons at the surface of the solid in contact with WiSE.

We now turn our attention to the electron on the left side of reaction (1a) above. For this purpose, we first consider the initial interaction of the X‐ray with our system (stainless steel/WiSE interface), which leads to excitation and ionization of the atoms in the solid and liquid, i.e.

(3) $$A\;\to \;A{}^{•}{}^{+}\;+\;e{}^{-}{}_{\mathrm{hot}}.$$

Here, A is either a water molecule, anion, cation, or an atom in the solid substrate, and electron‐deficient species A^.+^ is considered a “hole” (which in the case of the anions would be a neutral radical). This is schematically shown in Figure 3. The hot electron (e^−^_hot_), which carries excess kinetic energy after ionization, subsequently losses its kinetic energy via electron‐phonon mediated interactions with electrolyte species or substrate atoms (leading to further ionization in case of high‐energy electrons). The electron is considered thermalized when its kinetic energy equals the thermal energy (i.e. no excess kinetic energy from ionization) and is considered solvated upon solvent relaxation around the thermalized electron:

(4) $$e{}^{-}{}_{\mathrm{hot}}\;\to \;e{}^{-}{}_{\mathrm{therm}}\;\to \;e{}^{-}{}_{\mathrm{solv}}.$$

Hence, the thermalized electron is also often denoted pre‐solvated, which exists at higher chemical potential than the solvated electron, that is, it can be more reactive.^[21]^ We note that we assume here that the electron initially produced in the solid can easily overcome the work function of the substrate and has transported into the liquid during thermalization. Whether the thermalized or solvated electron will become a freely‐diffusing “free ion”, or whether electron‐“hole” recombination occurs, depends on the interplay between electron‐“hole” Coulomb attraction and thermal energy. In a scenario in which the electron can escape the Onsager radius (defined as the distance at which the thermal energy becomes larger than Coulomb attraction) during thermalization, the probabilities are high for free ion formation, and low for recombination. This depends on the dielectric constant of the solvent. Typical Onsager radii are 0.7 nm in water, 2.5 nm in ethanol, 20 nm in hexane, and approximately 1.5 nm in ionic liquids.^[22]^ Timescales for thermalization are around tens of femtoseconds in water^[23]^ and less than 1 ps in ionic liquids.^[24]^ Typical solvation timescales are on the order of ps for traditional low viscosity solvents (water and organic), whereas for ionic liquids with much higher viscosity they can be on the order of hundreds of picoseconds to tens of ns;^[25]^ here, we consider neat water the extreme case of *salt‐in‐water* (i.e. no salt), and we consider ionic liquids the extreme case of *water‐in‐salt* (i.e. no water). While little is known about the fate of electrons in WiSE upon interaction with ionizing radiation, it is reasonable to assume that time‐ and length‐scales in WiSE are between those of water and ionic liquids, that is, Onsager radii on the order of 1 nm, implying relatively large free‐ion yields, timescales for thermalization less than 1 ps (i.e. less than for ionic liquids), and timescales for solvation significantly longer than 1 ps. Assuming a linear scaling relationship between bulk viscosity and average solvation time, comparing the bulk viscosity and average solvation time in 1‐butyl‐1‐methylpyrrolidinium TFSI (95 MPa s, 270 ps^[25a]^) to the viscosity of 21 m WiSE (36 MPa s^[1a]^) leads us to speculate that electron solvation time in this system is on the order of 36 MPa s/95 mPa s×270 ps≈100 ps. The solvation time measured for photoelectrons in 3 monolayers of the same pyrrolidinium IL on Ag(111) was on the order of 60 ps.^[26]^ Approximate lower limit times for electrolyte decomposition reactions predicted from the QC calculations are typically greater than 0.1 ps, with the proposed reaction (1a) around 1–10 ps.^[17b]^ We thus postulate that the electron in reaction (1a) is a thermalized (pre‐solvated) electron, as it carries higher potential energy and can thus be more reactive.

The conjecture that reaction (1a) proceeds primarily through pre‐solvated electrons rather than solvated ones arises from threads of evidence from TFSI ionic liquids. Indeed, the 21 m WiSE electrolyte system can be considered a TFSI ionic liquid where the cation is defined as [Li⋅2.6 H_2_O]^+^. First, ab initio molecular dynamics simulations by the Margulis group^[27]^ showed that pre‐solvated excess electrons could attach to the TFSI^−^ anion and induce dissociation of the resulting dianion, in this case at the N‐S bond, within the first 50 femtoseconds. This pathway does not capture all excess electrons, since solvated electrons are observed in many aliphatic (non‐aromatic) ILs on longer timescales.[^22^, ^25^, ^28^]

Direct experimental evidence that lithium ions activate TFSI^−^ towards reaction with pre‐solvated electrons is depicted in Figure S9 of the Supporting Information. Figure S9 shows the decay kinetics of the solvated electron, measured by the decrease in its absorption at 900 nm, in neat Pyrr_1,4_TFSI and 0.66 m LiTFSI in Pyrr_1,4_TFSI. The traces are normalized to the same radiolytic dose, but the yield of solvated electrons at early time is 40 % smaller with 0.66 m LiTFSI than without. The “missing” electrons reacted in their pre‐solvated states with a complex activated by the presence of Li^+^ ion. Figure S9 also shows that the reactivity of solvated electrons with unbound TFSI^−^ anion is negligible, since that reaction would result in an exponential decay, while the observed decay is non‐exponential and driven mainly by recombination. Comparison of that decay with the scaled trace for 0.66 m LiTFSI (gray) shows that the solvated electrons decay slightly faster in the presence of Li^+^ ion, which can be accounted for by adding another decay term of *k*=1×10^6^ M^−1^ s^−1^ [Li]. For comparison, diffusion‐controlled solvated electron scavenging reactions in ILs similar to this one have rate constants on the order of 10^8^ M^−1^ s^−1^ or higher.^[22]^

The substantial formation and growth of LiF under the specific conditions of the WiSE/solid interface, and not (or to significantly smaller extent below our detection limit) in the bulk electrolyte, is likely due to a combination of factors. First, charges separated within the solid phase by the incident radiation tend to be mobile and migrate to the interface, where they cause chemistry to occur, thus directing additional reactivity to the liquid phase compared to the effect of radiation on the liquid itself. Second, the secondary electrons generated in the solid near the interface may escape recombination by thermalizing in the electrolyte, in a separate phase from their corresponding hole. This avoidance of recombination increases the radiolytic product yield near the interface. Consistent with this hypothesis, photoelectrons were observed to cause significant degradation of a thin film of 1‐butyl‐1‐methylpyrrolidinium TFSI on a Ag(111) surface.^[26]^ The degradation was attributed to dissociative electron attachment. Another possibility could be that the highly structured WiSE at the interface rearranges much slower than the bulk (as for example found for ionic liquids under confinement compare to bulk^[29]^), potentially leading to increased lifetimes of pre‐solvated electrons. If only pre‐solvated electrons participate in reaction (1a) because they are more reactive than solvated electrons, this explains why the reaction is only observed near the interface. These hypotheses and speculations should be subject to future studies.

## Conclusion

Using XCXP and various complemental techniques, we have observed that photoelectrons induced by X‐rays chemically reduce LiTFSI to LiF under *water‐in‐salt* conditions. The mechanism behind this reaction was proposed, for which interfacial contact ion‐pairs serve as the key ingredient, hence such reactions are only possible at high salt concentrations. As confirmed by QC predictions, this work shows that interfacial speciation determines interfacial chemistry on solid surfaces in *water‐in‐salt* electrolytes, and therefore might dictate the eventual electrolyte stability window. This finding also alerts one that caution must be exercised when using in situ or *operando* high intensity ionizing hard X‐rays to investigate the interfacial electrochemistry due to the overwhelming artifacts from XCXP reaction products. Using high energy X‐rays (>70 keV) might help to mitigate this issue.^[30]^ In this context, we also speculate that the observed radiation‐induced phenomena are not necessarily unique to *water‐in‐salt* electrolytes, but could occur in other highly concentrated electrolytes that contain fluorinated species (e.g. LiTFSI, lithium bis(fluorosulfonyl)imide (LiFSI), lithium hexafluorophosphate (LiPF_6_)) such as conventional non‐aqueous electrolytes,^[31]^ polymer electrolytes,^[32]^ as well as ionic liquid^[33]^ and gel electrolytes;^[34]^ some of these systems also exhibit LiF‐containing surface films. One the one hand, this might complicate certain in situ X‐ray experiments, but also opens the opportunity to study potential reduction reaction pathways via XCXP experiments.

## Conflict of interest

The authors declare no conflict of interest.

## Supporting information

As a service to our authors and readers, this journal provides supporting information supplied by the authors. Such materials are peer reviewed and may be re‐organized for online delivery, but are not copy‐edited or typeset. Technical support issues arising from supporting information (other than missing files) should be addressed to the authors.

Supplementary
