# Supplementary material for: Interfacial Speciation Determines Interfacial Chemistry: X‐ray‐Induced Lithium Fluoride Formation from Water‐in‐salt Electrolytes on Solid Surfaces
Source: Angew Chem Int Ed Engl. 2020 Oct 9;59(51):23180–7. doi: 10.1002/anie.202007745 (PMC7756515; doi:10.1002/anie.202007745)
Supplement: Supplementary file 1 — Supplementary [file ANIE-59-23180-s001.pdf]

Angewandte Chemie
Eine Zeitschrift der Gesellschaft Deutscher Chemiker

Supporting Information

Interfacial Speciation Determines Interfacial Chemistry: X-ray-Induced Lithium Fluoride Formation from Water-in-salt Electrolytes on Solid Surfaces

Hans-Georg Steinrück, Chuntian Cao, Maria R. Lukatskaya, Christopher J. Takacs, Gang Wan, David G. Mackanic, Yuchi Tsao, Jingbo Zhao, Brett A. Helms, Kang Xu, Oleg Borodin, James F. Wishart, and Michael F. Toney*

anie_202007745_sm_miscellaneous_information.pdf

1. Methods

X-ray chemistry-X-ray probe experiments (XCXP)[1] experiments were performed at beamline 2-1 at the Stanford Synchrotron Radiation Lightsource (SSRL) using 17 keV X-rays. The X-ray beam size was defined by a set of slits to 300 µm vertically and 1000 µm horizontally. The X-ray flux on the sample was ≈ 10^{11} photons/second, corresponding to a fluence of ≈ 3.3 e^{13} photons/second/cm^2. A Dectris Pilatus 100k area detector at 503 mm from the sample was used to collect scattered photons. The data was integrated into one-dimensional scattering patterns (intensity versus scattering vector **q**) using pyFAI[2]. The integrated intensity of the LiF (111) peak centered about q_{LiF111} = 2.702 $Å^{-1}$ was extracted as follows: First, a linear background was calculated and subtracted via a line through the average intensity between q ≈ 2.650 and q ≈ 2.730 $Å^{-1}$. Subsequently, the peak area was obtained by numerical integrating from q_{min} = 2.66 to q_{max} = 2.73 $Å^{-1}$. Finally, the extracted values were normalized by the average intensity of a region on the detector that contained only background scattering.

X-ray photoelectron spectroscopy (XPS) experiments were performed using the PHI Versaprobe 1 Scanning XPS Microprobe at the Stanford Nano Shared Facility (SNSF). The X-ray source is an Al K-alpha at 1486.6 eV. The pass energy was set to 23.5 eV. Charge neutralization was used. Peak positions were shifted by –1.4 eV after normalization to the S 2p3/2 level of $TFSI^-$ centered on 169.4 eV[3]. XPS peak integration for compositional analysis was performed using a PHI MultiPak 9.8.0.19. Scanning electron microscopy energy-dispersive X-ray spectroscopy characterization was carried out using a FEI Magellan 400 XHR scanning electron microscope. Optical microscopy was conducted on a Leica DM4000M microscope. Nuclear Magnetic Resonance (NMR) spectra were recorded on a Bruker Avance 500 MHz NMR spectrometer and processed using Bruker TopSpin 4.0.1 Software. Ex-situ grazing in X-ray diffraction was performed at beamline 11-3 at the Stanford Synchrotron Radiation Lightsource (SSRL) using 12.7 keV X-rays. The X-ray beam size was defined by a set of slits to 50 µm vertically and 150 µm horizontally. A Rayonix MX225 MAR CCD area detector at 250 mm from the sample was used to collect scattered photons during 30 seconds of illumination.

Pulse radiolysis transient absorption kinetics of solvated electron decays with and without LiTFSI in a TFSI ionic liquid were measured at the BNL Laser-Electron Accelerator Facility[4] using previously described methods[5]. Samples of pure 1-butyl-1-methylpyrrolidinium TFSI ($Pyrr_{1,4}TFSI$, IoLiTec) and 0.66 m LiTFSI (3M) in $Pyrr_{1,4}TFSI$ in septum-sealed 1 cm spectrophotometer cuvettes were purged with Ar to remove air for 30 minutes before the measurements.

For XCXP experiments, a solid-liquid interface scattering cell similar to the cell in Refs. [6] was used with a typical solution volume of 0.5 ml. The stainless steel (Grade 316, McMaster-Carr) substrates of size 4 x 30 mm were prepared by gentle polishing with ≈ 5 µm lapping film up to a mirror-like surface, and subsequently ultra-sonication in isopropanol prior to XCXP experiments.

Two aqueous electrolyte solutions were utilized. (1) 21 molal (m) LiTFSI in water; this is considered the *water-in-salt* (WiSE) case with 2.67 H_2O per LiTFSI. (2) An ≈ 1 m aqueous LiTFSI solution prepared by mixing the 21 m solution 5:1 per volume with water to ensure no effects from possible preparation contaminations; this is considered the *salt-in-water* case with 55 H_2O per LiTFSI.

Density functional theory (DFT) calculations were used to predict reduction potential for the $TFSI^-$ anion decomposition in the highly concentrated electrolytes. The $(Li_2TFSI(H_2O)_6)^+$ cluster was chosen a representative model with the water to Li^+ ratio similar to 2.67 as in 21 m LiTFSI. DFT calculations predict that this complex undergoes reduction around 2 V vs. Li/Li^+ either via the S-N bond breaking or defluorination and LiF formation as shown in Figure S9. After the $TFSI^-$ anion defluorination, formation of the C-C bond between the $TFSI_{(-F)}^{-}$• radical anions is highly favorable as shown in Figure S10.

Gaussian 09 software was used for all QC calculations.[7] A computationally less expensive global hybrid functional M05-2X with a compact 6-31+G(d,p) basis set was used for QC studies of the larger clusters after its accuracy was examined for the for smaller complexes via comparison with G4MP2 calculations. In addition to SMD solvation model using water (ε = 78) parameters, SMD(ε = 20) implicit water model were used to estimate the influence of solvation environment on reduction potentials.[8] Figure S10 shows that a dramatic change of the dielectric constant from ε = 78 to ε = 20 lowered reduction potentials by 0.2 V.

The reduction potential for the complex of interest denoted as complex A was calculated as the negative of the free energy of formation of A^- in solution [$\Delta G^S_{298}=G^S_{298}(A^-) - G^S_{298}(A)$] divided by Faraday's constant as given by:

$$E^{\circ} = -\frac{\Delta G^S_{298K}}{F} - 1.4\,V$$

The difference between the Li^+/Li and absolute reduction potential of 1.4 V was subtracted to convert results to Li^+/Li scale as discussed extensively elsewhere.[9]

2. Absorbed dose

Neglecting absorption by the electrolyte, the illumination corresponds to an absorbed dose D by the stainless steel substrate of $D = F \cdot E \cdot \frac{\mu}{\rho} = 3.5\ \mathrm{kGy/second}$, where F is the X-ray fluence of ≈ $3.3e^{13}$ photons/second/cm^2, E the X-ray energy of 17 keV, and $\frac{\mu}{\rho}$ the mass energy absorption coefficient of ≈ 38.5 cm^2/g for stainless steel (assumed composition is $Mn_2Cr_{17}Ni_{12}Mo_3Fe_{66}$) taken from;[10] integrated over 180 minutes this corresponds to 37746 kGy. This constitutes a large cumulative dose. In comparison, solvents used in nuclear fuel reprocessing may see a dose of ~500 kGy during their useful lifetime [11].

3. Experiments to test X-ray-induced LiF formation in bulk WiSE

While the bulk WiSE electrolyte significantly absorbs X-rays (1/e absorption length of ≈ 2 mm at 17 keV), we do not observe LiF formation in the bulk liquid. This was tested by exposing the liquid just above the surface to the X-ray beam for several hours, and subsequently measuring XRD of the surface below which would show LiF diffraction intensity for precipitated LiF. Attempts to investigate possible colloid formation in the irradiated WiSE solution by dynamic light scattering (after filtering with 200 nm PTFE syringe filter) suggested no presence of LiF colloids smaller than 200 nm. Experiments using unfiltered solutions were not reproducible and were hence inconclusive; we attribute this to particulate impurities in the sample, in

particular after irradiation, potentially due to impurities in the X-ray cell or the formation of soluble reaction products. The unfiltered samples were filtered through a 200 nm Watman AlO_x filter which was subsequently rinsed with ethanol (which solubilizes LiTFSI but not LiF). The filter was then investigated using grazing incidence X-ray diffraction (Figures S4) and XPS and no LiF/Li signal was found in the case of the irradiated WiSE solution (Figures S5 and S6).

4. Additional data

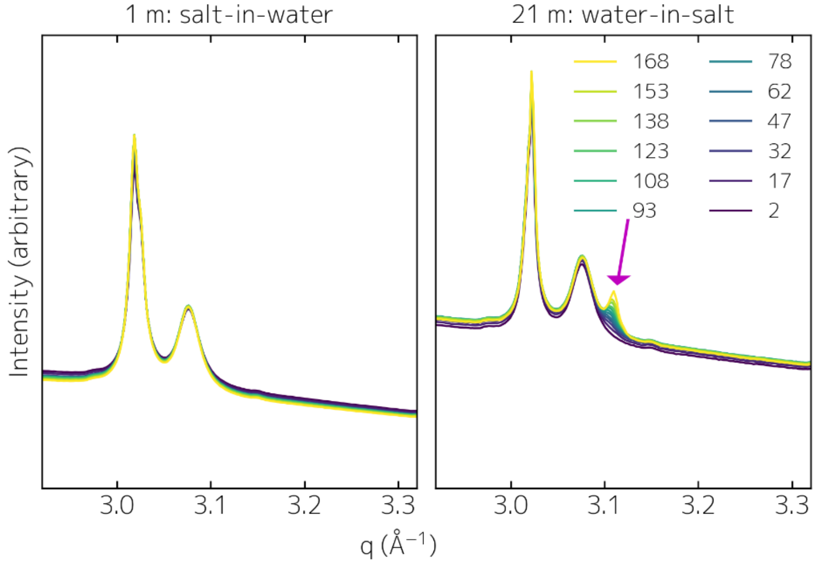

Figure S1: LiF (200) X-ray diffraction intensity as a function of X-ray exposure time of the electrolyte/stainless steel interface for the 1m salt-in-water solution and the 21m water-in-salt solution. Consistent with the results for the LiF (111) peak shown in Figure 1, the intensity remains unchanged in the 1m salt-in-water case, whereas it increases steadily with the exposure time (denoted in minutes in the legend) in the 21m water-in-salt case (as indicated by the magenta arrow).

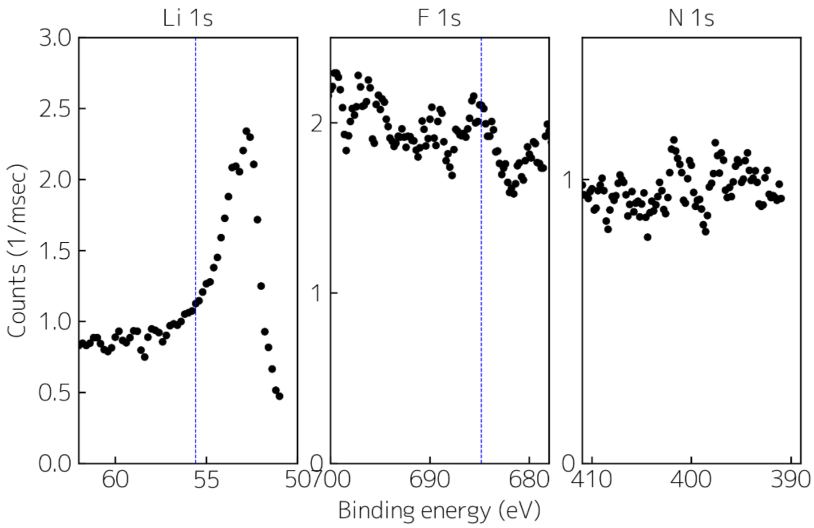

Figure S2: X-ray photoelectron spectroscopy (XPS) in the Li 1s, F 1s, and N 1S spectral range of stainless-steel after x-ray exposure in 1 m LiTFSI solution. The dashed blue lines correspond to the expected peak positions of LiF in the Li 1s spectral range (55.6 eV [12]) and F 1s spectral range (684.8 [13]). No evidence for LiF or left-over salt after washing is observed. The peak at around 53 eV in the Li 1s spectral range corresponds to the Fe 3p peak originating in the stainless steel electrode.

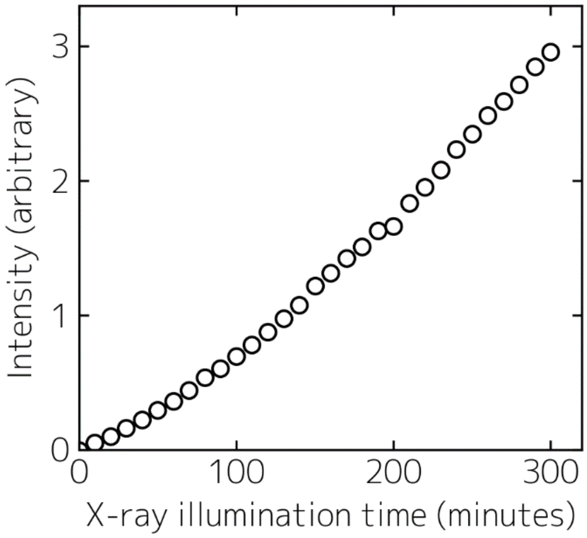

Figure S3: Integrated LiF (111) intensity as a function of X-ray exposure time of the electrolyte/sapphire interface for the 21 m water-in-salt solution.

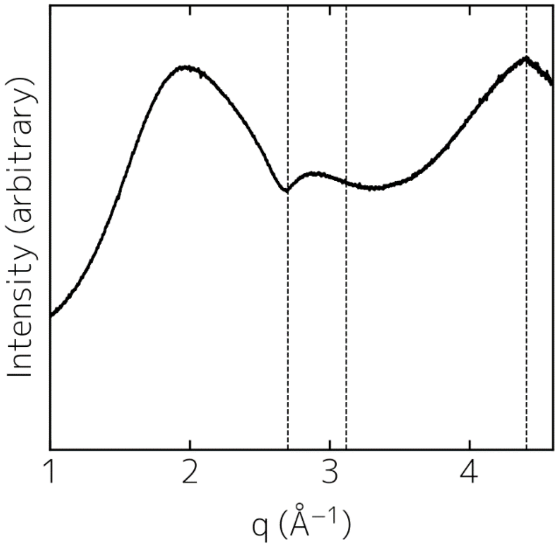

Figure S4: Grazing incidence X-ray diffraction of 200 nm AlOx filter after filtering of irradiated solution, showing no conclusive evidence for LiF. The dashed lines correspond to the expected peak positions for LiF ((111), (200), and (220) peak for increasing q).

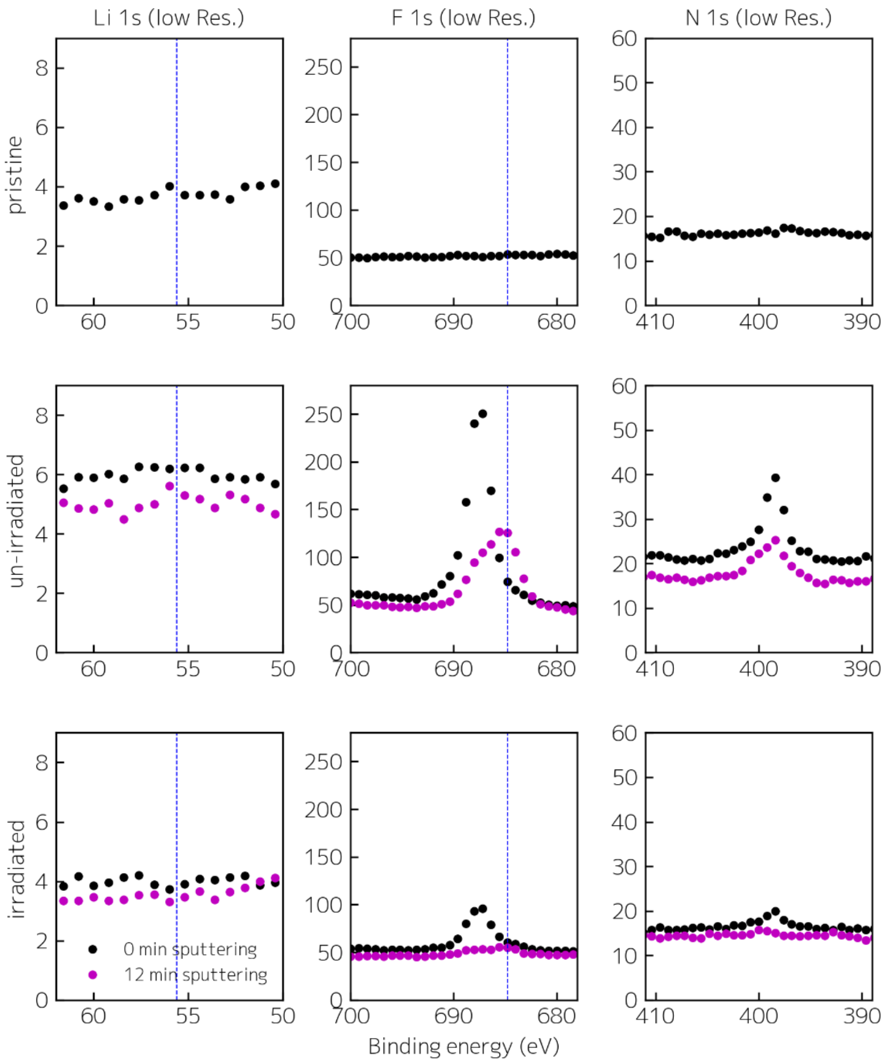

Figure S5: X-ray photoelectron spectroscopy (XPS) in the Li 1s, F 1s, and N 1S spectral range of pristine 200 nm Watman AlO_x filter (top), as well as AlOx filter after filtering of un-irradiated (middle) and irradiated (bottom) solution. All y-axes correspond to counts/ms. The dashed blue lines correspond to the expected peak positions of LiF in the Li 1s spectral range (55.6 eV [12]) and F 1s spectral range (684.8 [13]). The pristine and irradiated solution filtered filter show no detectable Li signal (see also Figure S6), which suggests that no radiation chemistry occurs to form LiF via reaction of an photoexcited electron with Li^+ and $TFSI^-$ in the bulk solution. Some left over LiTFSI after washing is present on the irradiated and un-irradiated solution as evident from the peak in the N 1s spectral range. This is consistent with a decrease upon ion sputtering. The un-irradiated solution shows some evidence for Li, even after sputtering, which may be consistent with some LiF (peak after sputtering in the F 1s at around 685 eV) or interaction of LiTFSI with AlO_x [14]; subtle changes in the Al spectral ranges (not shown) are also observed but a more detailed investigation is outside the scope of this manuscript.

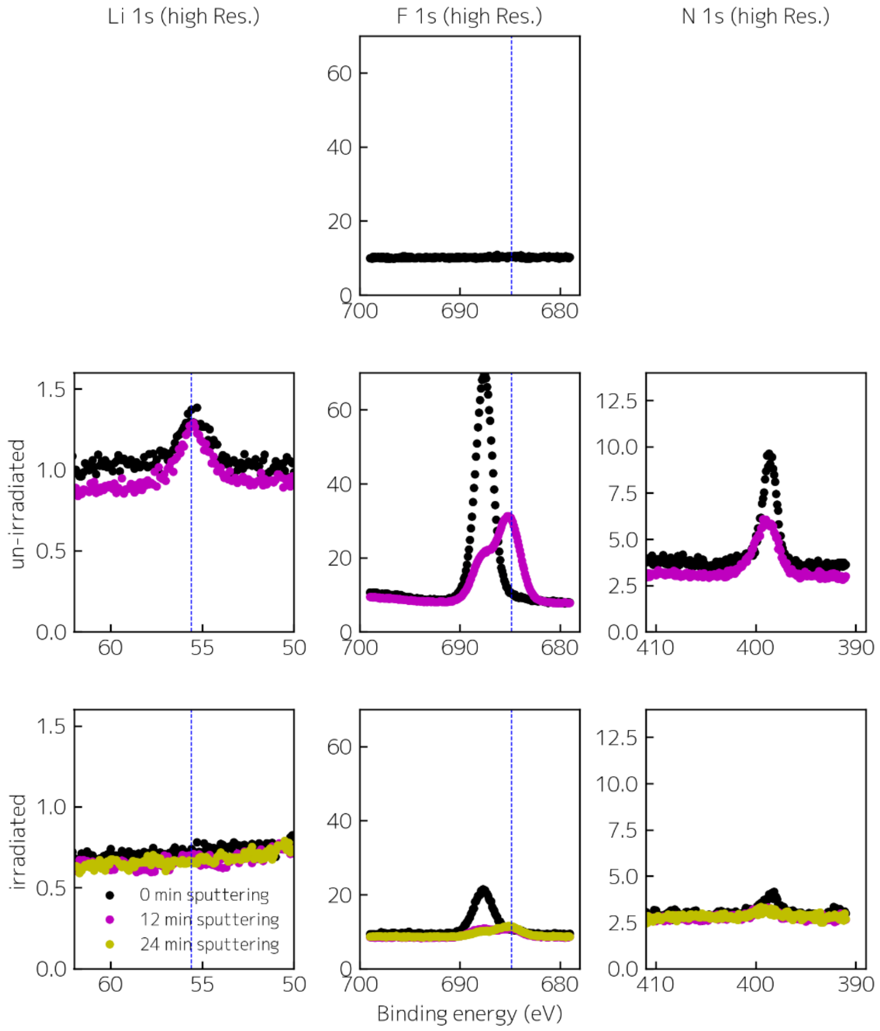

Figure S6: Same as Figure S5 but with higher data point density and longer averaging times to yield better statistics. Li 1s and N 1s was not measured.

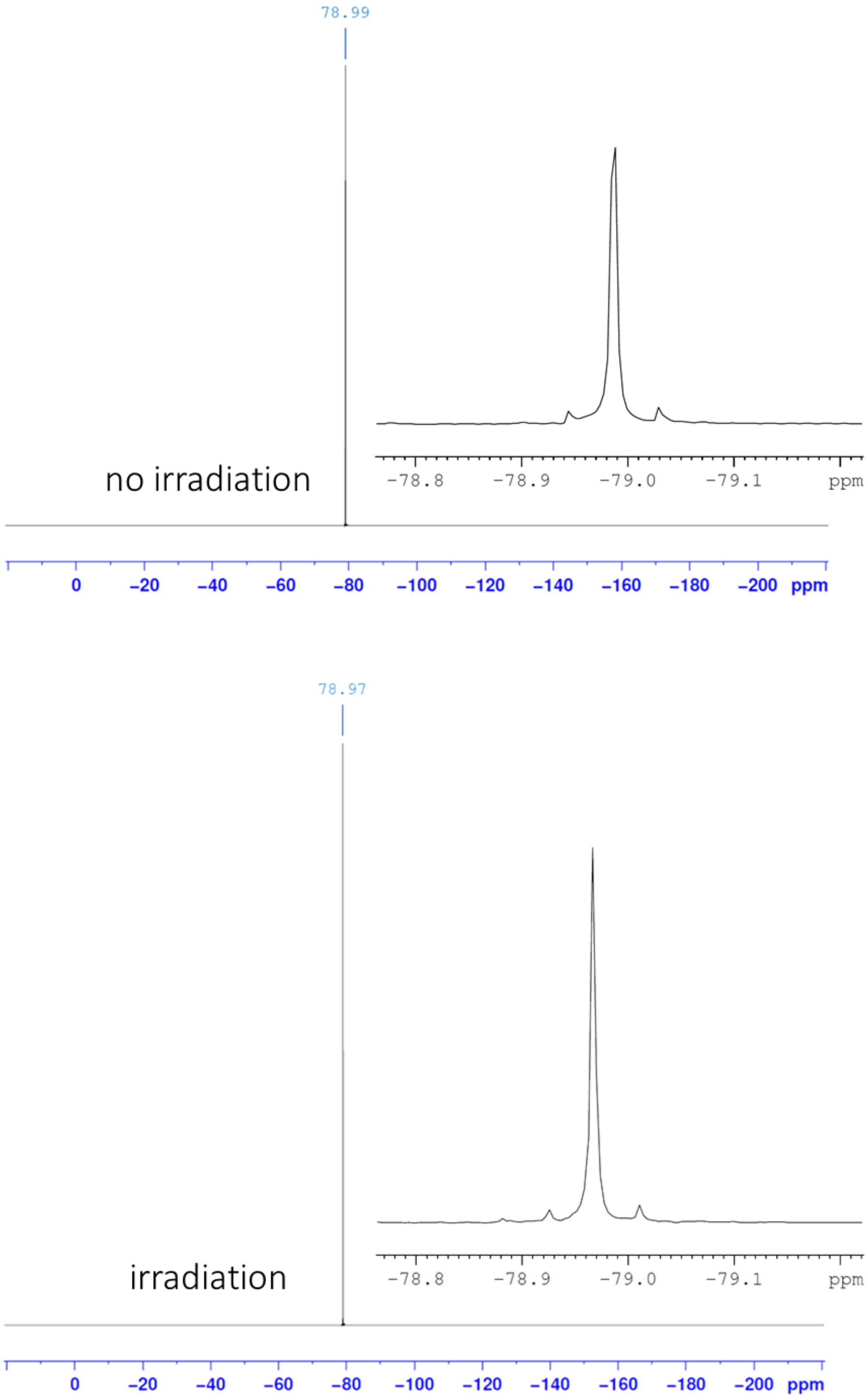

Figure S7: [19]F Nuclear Magnetic Resonance (NMR) spectra of 21 m salt-in-water solution without and with irradiation showing no CF_x signal.

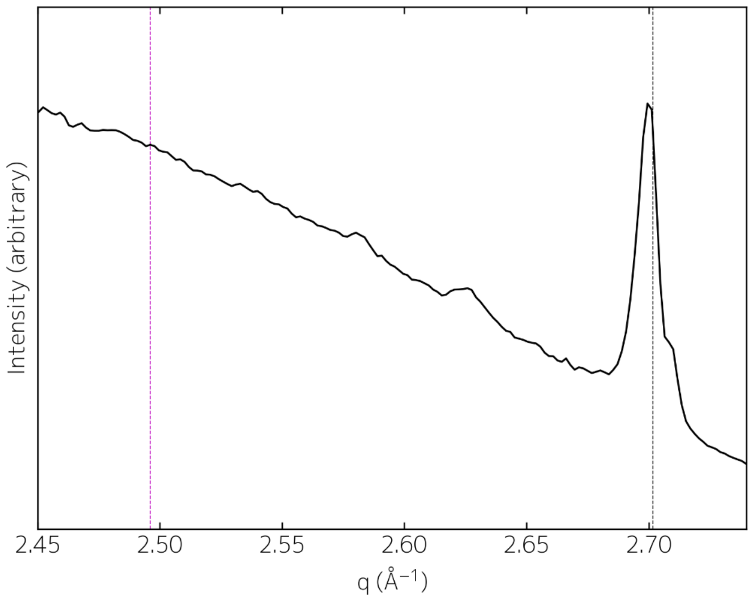

Figure S8: X-ray diffraction intensity after about 180 minutes of X-ray exposure of the electrolyte/stainless steel interface for the 21 m salt-in-water solution; magenta dashed line corresponds to the LiOH (110) peak position (no diffraction intensity observed) and black dashed line corresponds to LiF (111) peak position. The shoulder at the high-q side of the LiF (111) peak corresponds to signal originating in the electrolyte/Kapton window interface.

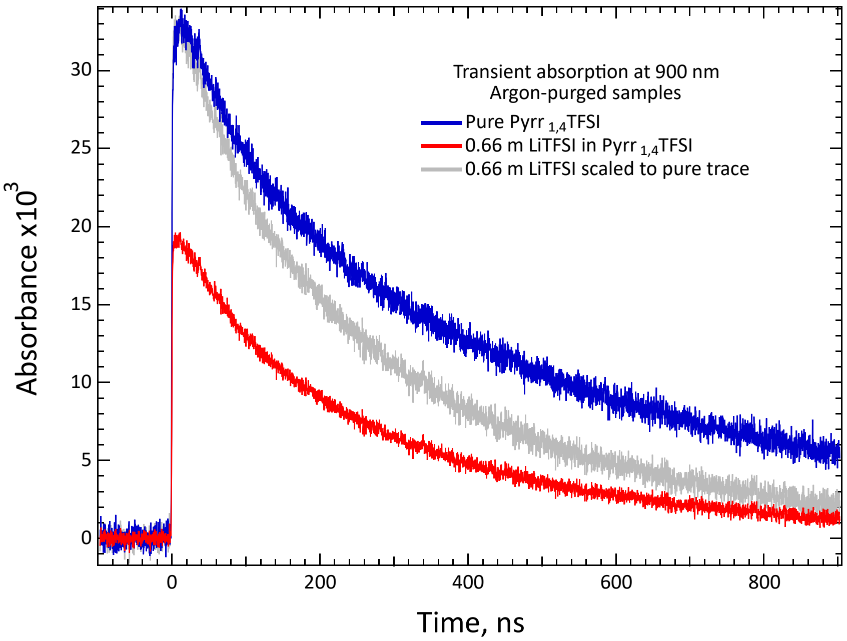

Figure S9: Pulse radiolysis transient absorption kinetics at 900 nm of pure 1-butyl-1-methyl-pyrrolidinium TFSI ($Pyrr_{1,4}$TFSI, blue) and 0.66 m Li TFSI in $Pyrr_{1,4}$TFSI (red). Grey trace: 0.66 m Li TFSI data scaled to the peak absorbance of the pure $Pyrr_{1,4}$TFSI trace. Pathlength 1 cm, dose 15 Gy.

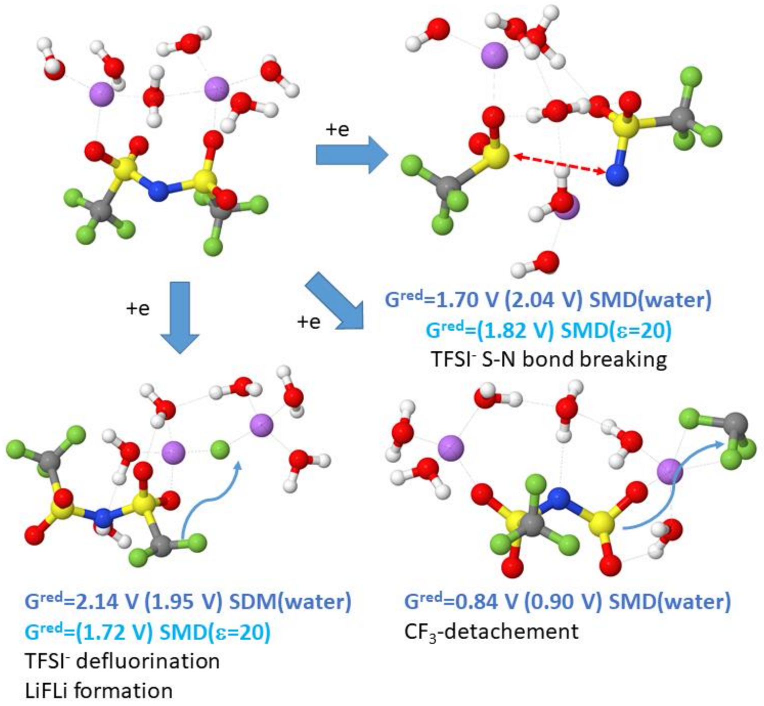

Figure S10: Reduction potentials for the $(Li_2TFSI(H2O)_6)^+$ complex (vs. Li/Li^+) from the more accurate but computationally expensive G4MP2 composite methodology and less computationally expensive M05-2X/6-31+G(d,p) (in parentheses) DFT calculations. Two implicit solvent model SMD(water) and SMD(ε=20) were used in calculations to estimate the influence of dielectric constant of the environment on reduction potential.

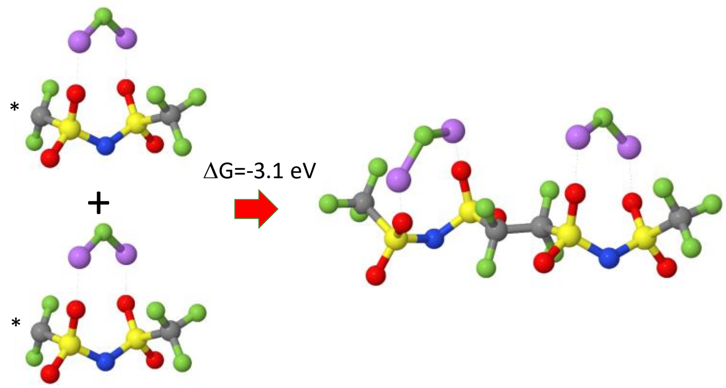

Figure S11. The C-C bond formation energy for the $TFSI_{(-F)}^-$• radical anions complexes with LiFLi from DTF calculations using M05-2X/6-31+G(d,p). The implicit solvent model SMD(water) was used in all calculations.

References

[1] M. Pravica, L. Bai, C. Park, Y. Liu, M. Galley, J. Robinson, D. Hatchett, *Rev Sci Instrum* **2012**, *83*, 036102.

[2] G. Ashiotis, A. Deschildre, Z. Nawaz, J. P. Wright, D. Karkoulis, F. E. Picca, J. Kieffer, *Journal of Applied Crystallography* **2015**, *48*, 510-519.

[3] R. Dedryvère, S. Leroy, H. Martinez, F. Blanchard, D. Lemordant, D. Gonbeau, *J Phys Chem B* **2006**, *110*, 12986-12992.

[4] J. F. Wishart, A. R. Cook, J. R. Miller, *Rev. Sci. Instrum.* **2004**, *75*, 4359-4366.

[5] J. F. Wishart, A. M. Funston, T. Szreder, A. R. Cook, M. Gohdo, *Faraday Discuss.* **2012**, *154*, 353-363.

[6] a) Y. Horowitz, H.-G. Steinruck, H.-L. Han, C. Cao, I. I. Abate, Y. Tsao, M. F. Toney, G. A. Somorjai, *Nano letters* **2018**, *18*, 2105-2111; b) H.-G. Steinrück, C. Cao, Y. Tsao, C. J. Takacs, O. Konovalov, J. Vatamanu, O. Borodin, M. F. Toney, *Energy & Environmental Science* **2018**.

[7] M. J. T. Frisch, G. W.; Schlegel, H. B.; Scuseria, G. E.; Robb, M. A.; Cheeseman, J. R.; Scalmani, G.; Barone, V.; Mennucci, B.; Petersson, G. A.; Nakatsuji, H.; Caricato, M.; Li, X.; Hratchian, H. P.; Izmaylov, A. F.; Bloino, J.; Zheng, G.; Sonnenberg, J. L.; Hada, M.; Ehara, M.; Toyota, K.; Fukuda, R.; Hasegawa, J.; Ishida, M.; Nakajima, T.; Honda, Y.; Kitao, O.; Nakai, H.; Vreven, T.; Montgomery, J. A., Jr.; Peralta, J. E.; Ogliaro, F.; Bearpark, M.; Heyd, J. J.; Brothers, E.; Kudin, K. N.; Staroverov, V. N.; Kobayashi, R.; Normand, J.; Raghavachari, K.; Rendell, A.; Burant, J. C.; Iyengar, S. S.; Tomasi, J.; Cossi, M.; Rega, N.; Millam, N. J.; Klene, M.; Knox, J. E.; Cross, J. B.; Bakken, V.; Adamo, C.; Jaramillo, J.; Gomperts, R.; Stratmann, R. E.; Yazyev, O.; Austin, A. J.; Cammi, R.; Pomelli, C.; Ochterski, J. W.; Martin, R. L.; Morokuma, K.; Zakrzewski, V. G.; Voth, G. A.; Salvador, P.; Dannenberg, J. J.; Dapprich, S.; Daniels, A. D.; Farkas, Ö.; Foresman, J. B.; Ortiz, J. V.; Cioslowski, J.; Fox, D. J, Gaussian, Inc., Wallingford CT, 2013, **2013**.

[8] A. V. Marenich, C. J. Cramer, D. G. Truhlar, *J. Phys. Chem. B* **2009**, *113*, 6378-6396.

[9] O. Borodin, in *Electrolytes for Lithium and Lithium-Ion Batteries, Vol. 58* (Eds.: T. R. Jow, K. Xu, O. Borodin, M. Ue), Springer New York, **2014**, pp. 371-401.

[10] NIST, *Photon Cross Sections Database* [*http://physics.nist.gov/PhysRefData/Xcom/Text*](http://physics.nist.gov/PhysRefData/Xcom/Text).

[11] L. Berthon, M.-C. Chabronnel, in *Ion Exchange and Solvent Extraction, A Series of Advances, Vol. 19* (Ed.: B. A. Moyer), CRC Press, Roca Baton, **2010**, pp. 429-513.

[12] K. W. Schroder, H. Celio, L. J. Webb, K. J. Stevenson, *Journal of Physical Chemistry C* **2012**, *116*, 19737-19747.

[13] G. E. Murch, R. J. Thorn, *J Phys Chem Solids* **1980**, *41*, 785-791.

[14] P. Jayathilaka, M. Dissanayake, I. Albinsson, B.-E. Mellander, *Electrochimica acta* **2002**, *47*, 3257-3268.
